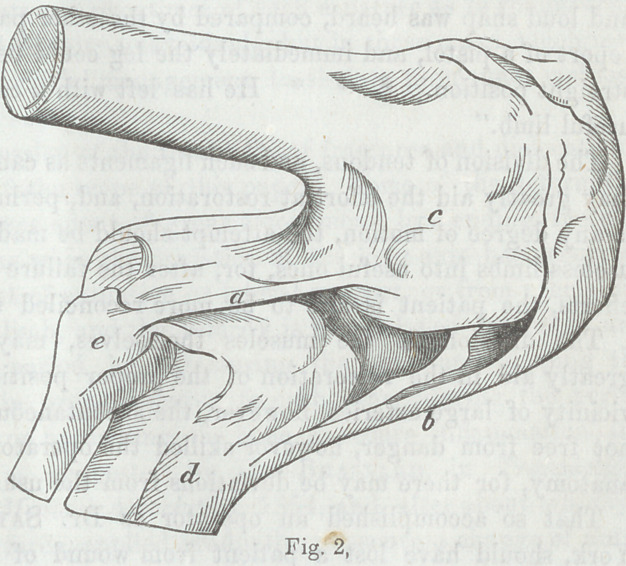# Report on Orthopedic Surgery, for the Annual Meeting of the Illinois State Medical Society, Held in Bloomington, May 2d, 1865

**Published:** 1865-08

**Authors:** David Prince

**Affiliations:** Jacksonville, Illinois


### CHICAGO MEDICAL EXAMINER.

N. S. DAVIS, M.D., EDITOR.

VOL. VI. AUGUST, 1865. NO. 8.

### REPORT ON ORTHOPEDIC SURGERY, FOR THE ANNUAL MEETING OF THE ILLINOIS STATE MEDICAL SOCIETY, HELD IN BLOOMINGTON, MAY 2D, 1865.

BY DAVID PRINCE, M.D., of Jacksonville, Ill.

[With 23 Illustrations.]

NOTE.—It is the aim of the following paper to discuss the subject of the nature and treatment of deformities to the extent of some degree of completeness, and yet as briefly as possible; and to simplify the processes so as to make them practicable with general practitioners of ordinary mechanical skill, who have no access to surgical instrument makers, and who must construct their own apparatus. Any detailed account of surgical operations is omitted, not because it would be irrelevant, but because it would extend the length of the paper too far. The subject of Talipes, having been fully discussed in the report of last year, is entirely omitted from the report of this year.

*Definition.*—ORTHOPEDICS, ORTHOPÆDIA, ORTHOPEDIC SURGERY—[From Gr. *orthos*, right, and *paidos*, a child.] The part of medicine whose object is to prevent and correct deformities in children. Used with a more extensive signification, to embrace the prevention and correction of deformities at all ages.

Prevention, in this, as in other branches of medicine, is far more important than correction, though usually less appreciated, because the extent of deformity obviated can only be estimated by those familiar with the subject. The impossibility of correcting many deformities, which by timely care might be prevented, adds still more to the necessity for a full account of the treatment of those diseases and injuries from which deformities result. The necessary limit of this paper has made a very full account of these lesions impracticable, but enough is said to indicate their character, and the sources from which fuller information may be derived.

The efficacy of time, skill, and patience in correcting malformations, is not only far beyond our popular credence, but beyond the estimate of the majority of the medical profession, who have not acquainted themselves with the recent advances in the art. The length of time often required is sometimes a source of discouragement with those who do not reflect by what slow and insidious processes deformities are often produced. The popular idea that surgery is cutting and curing quickly, and the impatience of surgeons themselves, who share this estimate of the art, are impediments in the way of the accomplishment of that degree of success which the general practitioners in every community should be able to secure.

PART FIRST.

The following classifications, by systematizing the conceptions, may aid in securing a clearer appreciation of the origins of malformations or malpostures, and of the means appropriate to their prevention and correction:—

*Classification of Deformities by their Causes.*

I. Arrest redundancy or misplacement of development. *Congenital.*

II. Perversion of relations of parts through muscular contraction, generally owing to palsy of the nerves distributed to one set of muscles, or to irritation of those distributed to their antagonists. *Con or post-genital.*

III. *Inflammation*, injuring or destroying the tissues, perverting or nullifying muscular contraction, distorting and stiffening joints, and perverting, impairing, or abolishing the functional movements of the parts affected. *Generally post-genital.*

IV. *Accidents*, breaking, tearing, bruising, burning, or freezing the tissues, followed by the loss of the parts, or the failure, imperfection, or perversion of their union or restoration. *Generally post-genital.*

V. Mutilation of parts designedly done; or force or restraint artificially applied, like the compression of the feet of the female children of Chinese grandees, or the heads of some Indian tribes, and, to a less degree, the feet and waists of genteelly educated children in modern American and European society. *Always post-genital.*

1. Arrest, redundancy, or misplacement of development. It is impracticable here to enter into a consideration of the varieties of congenital deformities, and only a few can be mentioned which admit of remedy. The most common of all these is hare-lip, which is an arrest of the process of union of the integument and other components of the upper lip to one side of the median line corresponding with the junction of the os maxillare with the os incissivum, which in man should become one bone, though they remain distinct in quadrupeds. The extension of the fissure between these two bones, and along the median line between the maxillary and palate bones, makes a fissure of the hard palate, and the still further extension through the soft palate, produces a cleft palate. The latter may exist alone while the lip and hard palate have their proper development. The union of opposite surfaces in the fissure of the lip is easily accomplished, that of the palate with more difficulty, while the union of the separated parts of the bony arch of the mouth has resisted the attempts of surgery, owing to the firmness of the bones of the face. Some diminution of the breadth of the fissure may have been secured by pressure upon the malar bones, and Dr. J. MASON WARREN, of Boston, has done something towards the closure of this opening by dissecting the tissues from the bony arch and causing them to *glide* backward to fill the posterior extremity of the fissure. [*See Am. Jour. Med. Sciences, Oct.*, 1863.] Various ingenious operators in dentistry have made admirable successes in closing the fissure of the hard palate by gold or hard rubber, but a more complete success has been achieved by NORMAN W. KINGSLEY, of New York, who has succeeded in closing the fissure in both the hard palate and the soft, with soft elastic rubber; and it is claimed that the expedient is quite as good as a closure of the soft palate by surgical operation. [*See Transactions Am. Medical Association,* 1864, *p.* 385.]

Hypospadias—an open state of the urethra from arrest of development, admits of some improvement by plastic operations securing artificial union, but here, and elsewhere, the defects must be supplied out of material sufficiently like the original to fulfil its function.

Redundancy, as in supernumerary fingers and toes, usually admits of an easy remedy in amputation, which should be so practiced as to leave the parts as nearly in their natural condition as possible; a point requiring no little surgical skill. Redundancy, in the uniting process between adjoining parts, gives rise to web fingers and toes. With the latter, encased in a shoe, the deformity hardly amounts to an inconvenience, but in the fingers the abnormal union is both inconvenient and unsightly. The fingers may be dissected apart, but the deficiency of integument is apt to lead to a reunion by a gradual encroachment of the fork of the fingers, very much like the process after a burn of the same parts. The sacrifice of the bones of a finger for the use of its integument to apply to the adjoining fingers, renders the operation more promising.

The misplacement of development not only constitute a very numerous variety of congenital deformities, but the most of them admit of no amendment. One limb is grown upon or into another, making one member, with all imaginable varieties; sometimes presenting the whole ten toes and fingers; there are sometimes two heads with one body, or two bodies, with their limbs, sustaining one head, or two distinct beings are united by more or less extensive connections. These rarely admit of any remedy.

A variety of misplacements occurs at a later period of development, depending upon mechanical restraints external to the fœtus. There is the greatest probability that solidifying exudations from the interior of the amniotic membrane may, for a variable period, take the place of the ordinary fluid secretion. Bands formed of this material may, in some cases, produce amputation; in others, fissures, in some instances dividing muscles, leaving the parts to be united by tendinous connections, and in other instances producing various malpostures of the limbs, among which may be some varieties of talipes. In many of these cases, properly applied force, after birth, may remove the malposition, while fissures may be obliterated by surgical operations.

2. Perversion of relations of parts through muscular contraction, generally owing to palsy of the nerves distributed to one set of muscles, or to imitation of those distributed to their antagonists.

The admission of the existence of the same kind of deficiencies, or perversions of the nervous function in intra and extra-uterine life, throws much light upon many congenital deformities. While talipes probably often owes its existence to some constraint, analogy leads us oftener to entertain the theory of irregular muscular action, through disease of the nervous system. The frequency of hydrocephalus during intra-uterine growth, shows that the fœtus is liable to the same perversions of nutrition which in childhood produce the same pathological results. For illustration, a contraction of the little finger most, and of the other fingers less, while the fontanelle is seen to be depressed, is sometimes observed as a symptom of the incipiency of those cerebral lesions in insufficiently nourished children, which, when unchecked, end in convulsions and death. A prolongation of this incipient state, previous to birth, or subsequent to it, might easily be supposed to produce such a preponderance of the flexors over the extensors as to require artificial aid to secure restoration.

A change of the state of nutrition by proper alvine evacuants, and an increase or alteration of the nutriment may, in children, remove the cerebral irritation, and relieve the local tonic spasm before the affected muscles become permanently shortened. On the other hand, an inflammatory excitement may have an equally abortive, protracted, or fatal course, with equally varied effects upon muscular contraction. Sometimes these lesions of the nervous system are so local as not apparently to pervert the general health, and yet the proper general treatment often removes the irritation and corrects the abnormal muscular contraction. In children, the progress of these cases can be observed, but in the fœtus the nature of the lesion can be known only by the result.

The interest and importance of this subject justify its consideration to a greater extent. Its correct understanding is necessary to the adoption of such therapeutics as will *prevent* deformity or loss of function, which is far better than the greatest success in restoring them. Any discussion on the subject of orthopedia which should omit this, would be defective in one of the most important points. A correct theory of the nervous irritation has two practical ends—first, to subdue or ameliorate the original nervous disorder; and, second, to preserve the functions of the parts receiving the perverted nervous stimulus so that when the original disease passes by, the local parts shall not have become irredeemably useless by loss of nutrition, or the assumption of malpostures, or both combined.

Great light is thrown upon this subject by the hypothesis, pretty well sustained, that there may be a constant muscular pull in addition to the ordinary muscular tonicity, and independent of, and sometimes in spite of, and in antagonism to voluntary contraction and ordinary reflex action. This constant abnormal contraction results in shortening, rigidity, and ultimate wasting of the muscles affected, reducing them to the condition and function of ligaments—in the language of BARWELL, the condition of *contracture.*

This is something more difficult to control than mere palsy or loss of action of certain muscles, leaving their antagonists to distort the parts affected by a mere excess of tonic and voluntary power. One of the best and most recent surgical writers in the English language has, however, thrown doubt over this hypothesis, or been disposed greatly to narrow its field. We quote from *Barwell on Club-Foot, p.* 19. (Churchill, London, 1863.) "Infants are frequently, as is well-known, subject to convulsions, and it is averred that sometimes one or more which have, during the attack, drawn the limb into malposture, do not recover from the contraction, but continue to keep the limb distorted. Such a muscular condition would not be a *contracture*, as I have described it, since the convulsion never lasts sufficiently long for any such organic process of areolar shortening; the state would be one of persistent, unvarying spasm, powerful enough to overcome the antagonistic healthy muscles, and permanent enough to produce lasting change of form. Such condition does not only never come under our notice, but is, I believe, pathologically impossible. There are, no doubt, a few cases of paralysis of voluntary power over the muscles, while the excito-motory function continues, and in the spasm of the whole set, the strongest will, of course, predominate. Voluntary power is as much used to control as to excite. The paralysis of this power is evidenced as much by violent and uncontrollable spasm, as by insusceptibility of subordinate movement."

The hypothesis in question is in perfect accordance with the last part of this quotation—maintaining that in addition to the excito-motory function, there is another which does not tire during the continuance of the irritation, but constrains the muscles affected to draw continually, while the capillaries are so squeezed as to receive an inadequate supply of blood, resulting in rapid wasting of the muscular substance, from want of nutrition. In illustration of this subject, the following quotations are from Dr. R. B. TODD'S account of paralysis with rigidity. (*Todd on the Nervous System. Lindsay & Blackiston's Edition.* 1855. *Page* 153.)

After speaking of paralysis, with relaxed muscles, he says:— "I mentioned that the second kind of hemiplegia is where the muscles of the paralyzed limbs are rigid, and where the rigidity comes on simultaneously with the paralysis, or very soon after it. You must bear in mind that a distinctive feature of this hemiplegia is the early period at which the muscles assume this rigid condition. This is the more important, inasmuch as we meet with another form of hemiplegia with rigid muscles, in which the stiffness gradually supervenes a long time after the paralytic seizure, and may succeed to the relaxed condition of the paralyzed muscles."

*Page* 155. "The condition of slight and partial rigidity of paralyzed muscles is that of most frequent occurrence in hemiplegia caused by an apoplectic clot. My idea as to its cause, is that it depends upon a state of irritation, propagated from torn brain to the point of implantation of the nerves of the affected muscles. But you will ask, Why is it that, in some cases of clot, the hemiplegia will be accompanied with complete relaxation of muscles, while in other cases the rigidity of which I have spoken exists? The answer to this question is as follows:—In the cases where there is no rigidity, the clot lies in the midst of softened brain, and has not in any degree encroached upon sound brain, but when rigidity exists, the clot has extended beyond the bounds of the white softening, and has torn up, to a greater or less extent, sound brain."

*Page* 158. "A much more interesting form of the second kind of hemiplegia, is that in which there is considerable rigidity of all the muscles of the arm and forearm, where the arm is kept at an angle with the trunk (and sometimes these patients hold it across the chest), the forearm being flexed on the arm, and the fingers flexed on the palm. In these cases the paralyzed muscles seem to be firm and contracted, and sometimes almost in a tetanic state, and offer considerable resistance to flexion or extension, which frequently also excites a good deal of pain. When the rigidity is of this nature, the paralysis is generally not complete, a certain degree of power remaining of moving the whole limb, or some part of it, and very frequently sensibility is affected, being sometimes obtuse, but oftener in an excited condition, while it not uncommonly happens that reflex actions are also considerably exalted. In the latter cases, you will find the reflex actions produce a considerable pain."

*Page* 159. "When hemiplegia, with a rigid state of the muscles, supervenes soon, on some injury of the head, you may almost always make a certain diagnosis that it is the result of irritant compression of the opposite hemisphere, by depressed bone or hemorrhage, outside or inside of the dura mater."

*Page* 160. "It (paralysis with rigidity) is due to a cause which exercises, at once, a paralyzing and irritating influence on the brain, and this influence is propagated to the spinal cord, and through the nerves implanted in that portion of the nervous centre, to the muscles of the paralyzed limbs, in which it excites a state of contraction. The effect is analogous to that produced by the continued action of the electro-galvanic machine, and just as a rapid succession of electric shocks may keep up this rigid condition of muscles, so may continuous shocks of nervous force, due to irritative pressure, bring about a similar result."

*Page* 178. "I think it must be admitted that the rigid state of the muscles is due, primarily, to an irritated or excited state of the nerves, and, that on the cessation of that irritation, the muscles might resume their relaxed condition, or that a similar result would follow a severance of all connection between the muscles and the seat of the cerebral lesion, by section of the nerves. It seems to me, however, that after a long continuance of this rigid and shortened state, the muscles would become permanently shortened, and would assume a condition similar to that into which anchylosed joints are apt to fall—a condition from which they would recover very slowly or not at all."

These detached quotations necessarily appear dogmatic, and for the facts and reasons through which the conclusions are arrived at, the reader is referred to the book from which they are taken.

BROWN-SEQUARD goes a step farther, and claims that this permanent contraction may occur, not only from irritation at the origins of the nerves in the encephalon, but that it may occur from irritation of the appropriate nerves in their course from centre to circumference. He is of opinion that there are nerve-fibres going from the brain to the muscles, the irritation of which produces tonic or permanent contraction of the muscles to which these nerves go, and that these nerve-fibres pass down in portions of the cord different from those through which the voluntary nerve-fibres pass. A few quotations are here introduced from the valuable work of this author. (*The Physiology and Pathology of the Nervous Centres, p.* 194.)

"The division of these nerve-fibres is not followed by paralysis, although they are able to act on muscles to produce contractions, and even more powerful than those caused by nerve-fibres employed by the will in voluntary movements."

"It is a fact, worthy of attention, that a puncture with a needle through the anterior pyramids which contain very nearly all, if not all, the nerve-fibres employed in voluntary movements, will hardly produce a momentary contraction in some muscles, while certain punctures through the olivary column of the medulla oblongata at once produce a spasm of many muscles, although this column does not contain more than very few voluntary motor-fibres, if any at all; and now, to add to the strangeness of the fact in this last case, the muscles remain contracted for hours, sometimes for days and weeks."

"We have all been taught that after the removal of a cause of excitation in the nervous centres, as well as in the nerves, the effects of the excitation disappear, until inflammation supervenes and produces a permanent excitation; while here, however, we see a puncture with a needle, or a section with a knife, before any inflammation can have begun, followed by a persistent effect. There is, therefore, in some parts of the nervous centres, a property of acting in a persistent manner to produce muscular spasms during, and after a mechanical excitation.

*Page* 196. "The parts of the base of the encephalon which are capable of producing persistent spasms, seem to be quite different from those employed in the transmission of sensitive impressions, or of the orders of the will to the muscles, at least in the medulla oblongata and pons Varolii. They constitute a very large portion of these two organs, and perhaps three-fourths of the first one. They are placed chiefly in the lateral and posterior columns of these organs; many of their fibres do not decussate and produce spasms on the corresponding side of the body; they seem to contain most of the vaso-motor nerves, by which, directly or through a reflex action, they may act on other parts of the nervous system. They have much to do with the phenomena of several, if not most of the convulsive diseases. Lastly, I will say that the history of their properties and actions throws a great deal of light upon the effects of disease or extirpation of the cerebellum."

*Page* 207. "There are a great many nerve-fibres and nerve-cells in the medulla oblongata, the pons Varolii, and other parts of the base of the encephalon, which are not employed in the transmission of sensitive impressions, or of the orders of the will to the muscles, and are endowed with the singular property of producing, even after a slight irritation, a persistent spasm in certain muscles, and especially in the neck."

Accompanying this rigidity of muscles from permanent spasm, there is supposed by this physiologist to be a similar excitement of the nerves going to the bloodvessels of the same muscles, by which the smooth muscular tissue of their walls is made inordinately to contract, depriving the affected muscles of an adequate supply of blood, and producing a rapid atrophy, while a similar irritation going to the voluntary muscles, secures their continued rigidity. It has been a mystery, how muscles could be actively contracting and yet rapidly wasting, and the hypothesis of BROWN-SEQUARD affords a plausible explanation of this feature. He further says, *p.* 163:—"That the paralysis of atrophied muscles is not the only cause of atrophy, is shown by the fact that this state (paralysis) of the muscles has often existed without paralysis, or at least before paralysis, and, sometimes, although there were convulsions in the muscles. NOTTA mentions three cases in which there were constant or frequent convulsions while atrophy was progressing."

Dr. BROWN-SEQUARD holds the theory that the paralysis of the voluntary muscles may, in some cases, be itself produced by irritant contraction of the muscular substance of the capillaries supplying with blood the origins in the brain or spinal cord of the nerves implicated. Dr. S. WEIR MITCHEL, of Philadelphia, objects to this, that this explanation cannot be true, because the spasmodic contraction of the capillaries must relax. If it is admitted, however, that there may be permanent spasm of the voluntary muscles, it is not difficult to conceive the existence of such a state of muscles that are involuntary.

Dr. MITCHEL'S views are explained, at considerable length, in the *American Medical Times, July* 9, 1864, and in the *American Journal of Medical Sciences, Jan.*, 1865, *p.* 161.

Dr. BROWN-SEQUARD'S theory finds further support in the fact that in paralysis with a flaccid condition, atrophy can, to a considerable degree, be obviated by passive motion, and in this connection the following quotation has great interest:—

*Page* 177. "Most of the morbid changes which have been attributed to paralysis do not belong to it, but are the result of irritation, either upon the nervous centres or upon the nerves, and the effects which are truly the consequences of paralysis, are due only in an indirect way to the absence of nervous action. Atrophy of muscles is chiefly due to a state of rest; changes of nutrition are chiefly due to dilatation of bloodvessels; ulceration upon the toes of animals, in which the nerves of limbs have been divided, only show the effects of rubbing the same parts upon the floor; ulceration and inflammation of the eye, after section of the trigeminal nerve, are chiefly due to physicauses (the drying of the cornea and conjunctiva, the prolonged action of light, &c.) All these may be, and have been, sometimes avoided.

"On the other hand, if we try to find out what is the power of cicatrization and repair, in cases of paralysis not complicated with irritation of nerves, we ascertain, as has long ago been done by Sir BENJAMIN BRODIE, and as we have done since, and in varying more the mode of experimenting, that wounds, burns, and fractures may be cured as quickly in paralyzed parts as in others. If the influence of the nervous system is indirectly necessary to nutrition and secretion, it is, nevertheless, true that all the phenomena of nutrition and secretion may remain normal, when the action of the nervous system on the various tissues is missing."

The irritations which give rise to palsy of voluntary muscular action, with the rigidity from irritative action of the involuntary nerves going to the same muscles, need not arise from irritating causes seated permanently at the origins of the nerves affected, but may be reflected from some peripheral seat of irritation. This affords not only ground of hope for relief, but a hint at the expedients of treatment.

*Treatment.*—BROWN-SEQUARD thinks from his own experiments, and those of MATTEUCCI, that certain contractions of muscles, from irritations derived through the nerves from their centres, there is the condition of cramp. It is found that in contractions of this nature, the pain is increased by the elongation of muscles, and entirely relieved by their complete relaxation, either by the approximation of their attachments, or by tenotomy. He says, (*loco citato*, *p.* 8):—"Now, I have found that the greater is the resistance to the contraction of a muscle, the greater is the galvanic excitation that it gives to the nerves in contact with its tissue. On the contrary, if there is no resistance at all, as shown by Prof. MATTEUCCI, after the section of the tendon, the galvanic excitation of nerves in contact with the contracting muscle no longer exists. It is not necessary for a muscle to contract in order to produce in nerves in contact with it a galvanic excitation. It is sufficient that it tends to contract."

One of the experiments referred to, consists in preparing a leg of a frog, with its sciatic nerve laying upon the muscles of another limb. The latter muscles are galvanized, and the first muscles are excited through the sciatic nerve. It is found that when there is no resistance to the contraction of the latter muscles, there is no galvanic excitation—no motion of the first muscles.

(Fig. 1.)

Fig. 1. From BROWN-SEQUARD.

*m* A muscle made tense by the attachment of a weight *w*.

$m^2$ Another muscle with nerve *n* lying upon the first muscle. When the muscle *m* tends to contract under the stimulus of galvanism, being restrained from movement by the weight *w*, the muscle $m^2$ contracts by galvanic excitation acting through its nerve $n^2$ which is distributed to the muscle $m^2$ of the other limb.

It follows that in cases of pain, in consequence of the contraction of muscles, as in cramps and rigidity, the division of the tendons of the muscles obviates the pain, by rendering null the galvanic excitation. This physiological explanation is also applied to explain the relief afforded by division of the sphincter in *fissura in ano*.

If the irritation causing the contraction were a permanent element, there would be little good obtained by dividing tendons; for with the accommodation of the muscular fibres to their shortened relations as the ends become again fixed, the painful contraction would be reproduced. It is only when the irritation derived through the nerves is to some extent temporary that permanent relief should be expected from this expedient.

In case of anal fissure, the ulcer may itself be the cause, by reflex action, of the painful contraction of the muscle. If the division of the muscle permits the ulcer to heal, the cause of painful reflex contraction is removed; and after the restoration by granulating cicatrization, of the connexion between the divided portions of the sphincter, the muscle again quietly performs its function.

The rational of treatment, here explained, throws a clearer light upon the benefits claimed by GUERIN and others, from the division of muscles in distortions of the body and limbs with a condition of the contracting muscles resembling a permanent spasm. This explanation may also lead to a clearer distinction between those cases which can be benefited by division of muscles and tendons and those which cannot.

In those disproportionately contracting muscles which escape the wasting process a true hypertrophy occurs, and this continues after the irritating cause has disappeared. In these cases the division of muscles and tendons must hold out less promise; for in a short time the wound must be cicatrized, and the intervening material must contract like cicatricial substance elsewhere, restoring the muscle to its original length and power.

Another plan of treatment may here be applied—that of extension, to the extent of exhausting and gradually lengthening the shortened muscle.

In considering the philosophy of the influence of extension in producing the elongation of muscles, the analogy of the paralysing effect of our distension of the muscular fibres of the uterus and of the bladder is in point. As long as the increased length of muscular fibres is maintained they manifest very little power of contraction. But the partial evacuation of the contents by the rupture of the membranes, and the escape of the amniotic fluid, or, in retention of urine, the free flow of the urine through a catheter, restores the muscular fibres to the conditions of activity, and they proceed in a little time to discharge their proper functions, or if maintained by a definite increased length they soon accommodate themselves to the new conditions, and acquire the capability of contraction and relaxation in the increased distance.

It is obvious then, that in the treatment of deformities resulting from the contraction of muscles, the conditions of the muscular fibres of an over-distended uterus or bladder may be advantageously produced by artificial means. When the fibres have been drawn out, or induced to grow out of the desired length, the diminution of extension permits the muscular fibres to assume the capabilities of the elongated muscles sometime after a dislocation. It is obvious, from these considerations, that while a very moderate force would only increase the power of the fibres in a given length, a greater degree of force would increase the length with temporary diminution of power. On the other hand, the extension of the shortening or shortened muscles diminishes the acting length of the yielding antagonist muscles, and permits them quickly to acquire increased power. It is not necessary that they should acquire the same power as their overgrown antagonists, for we know that flexors and extensors are rarely balanced; and if it were the nature of the stronger muscles to overbalance the weaker what grotesque shapes should we all very speedily assume.

It is not the true indication permanently to weaken the shortened or hypertrophied muscles, but to accustom them to normal positions and limitations of contraction; automatic and voluntary nervous action must be relied upon to control the tendency of unequal powers of unequal muscles to produce distortion by the tonicity of health. Until this habit is complete and lasting, the artificial restraint to the strong and support to the weak must be employed. It is from the premature discontinuance of the mechanical appliances to this end, that disappointment is too often due. In many cases it is necessary to resist, not only the tonicity of health, but the tendency to rigidity arising from nervous irritation; and, while this tendency lasts, the mechanical appliances must be worn. To obviate deformity is both wiser and more easy than removing it, when it has once occurred.

For paralysis of muscles without tendency to rigidity, on obvious principle of treatment, is to secure such artificial movements as may most nearly imitate the natural. By this means the circulation through the capillaries is kept free and nutrition may go on; bones unite and wounds and burns heal in parts destitute of both motion and sensation if only by motionlessness the capillary circulation does not fail.

Where the cause of the paralysis is temporary the great labor necessary to secure this artificial activity may meet its full reward in ultimate restoration of the power. It is not general exercise that is here so much needed but "localized movements," which must be performed by a third person, or by some kind of mechanism, a good idea of which will be obtained by reading Dr. C. F. TAYLOR'S *Movement Cure.*

*Electricity.*—There never was greater discrepancy of opinion among practical men upon any subject, than exists among physicians upon the use of elcctricity in deteriorations of muscles, or perversions of their functions. It is suspected that this has grown out of the want of proper distinctions.

In proof of the power of electricity to preserve the volume of paralyzed muscles, nothing can be more conclusive than the experiment of Dr. JOHN REID, which I quote from *Althaus on Medical Electricity, p.* 143.

"Dr. REID cut the spinal nerves in the lower parts of the spinal canal in four frogs, so that both hinder extremities were insulated from their connection with the spinal cord. He then daily exercised the muscles of one of the paralyzed limbs, by a weak galvanic current, while the muscles of the other limb were allowed to remain quiet. This was continued for two months, and at the end of that time, the muscles of the galvanized limb retained their size and firmness, and contracted vigorously, while those of the quiescent limb had shrunk to at least one-half their former bulk, and presented a marked contrast with those of the galvanized limb."

The form of electricity employed for the purpose of preventing muscular atrophy, or stimulating muscular development, when they are deprived of their appropriate nervous stimulus, is the interrupted current of an *induction* apparatus, either deriving its electricity from the decomposition of metals, as the ordinary galvanic battery, or from the magnet, as in the magneto-electric machines. The continuous current is found to have no power in producing contraction of voluntary muscles, though a slight contractile effort is produced upon smooth muscular substance, though these latter respond more readily to the interrupted than to the continuous current. In the language of *Althaus, p.* 177:—"The contractions are observed, not only at the commencement and the cessation of the current, as is the case with the voluntary muscles, but also while the circuit remains closed. * * * The movements induced in the muscular fibre cells by galvanism, are not observed simultaneously with the application of the electric stimulus, as with voluntary muscles, but only a certain time after the current has acted upon the tissue. * * * Besides, the motion once excited in the fibre cells, continues a certain time after the cessation of the current, and is not confined to those parts to which the electric current has been directly applied, as is the case with the voluntary muscles, but is propagated to other parts of the same tract."

It is found that a continuous current of electricity, as well as an interrupted current, secures in the now striped or smooth muscular fibres of the vessels, a contraction which lasts a considerable time after the cessation of the current. The effect is to contract the bloodvessels which come in the course of the current, diminishing the amount of blood which they contain, and, consequently, diminishing the temperature.

Upon the voluntary or striped muscles, however, the contraction is immediate upon the beginning or cessation of the current, and ceases immediately and until a new excitation by the beginning or cessation of a current. It follows, that the application of electricity to a muscle produces two effects antagonistic to each other. First, a contraction, followed immediately by a relaxation of the muscular fibres, by which blood is invited to the tissues, partly by the change of volume increasing and diminishing in alternation, and partly by the chemical change of the elements of the tissues, under the physiological action of the muscle. By this increase of blood, the temperature rises, and the nutrition and volume of the muscle are maintained. Secondly, owing to the tendency of the vessels at the same time to contract, a certain intensity is given to the rapidity of the flow of blood, which resembles that imparted by the presence of moderate cold. The continuous current acts very slightly upon voluntary muscles, but quite considerably upon smooth muscular fibres, which give contractility to the walls of the vessels. Therefore, the interrupted current should always be employed to secure a better nutrition of voluntary muscles.

In the state in which there is a rigidity or tonic contraction of the muscles, owing to irritation of nervous centres, the application of electricity would seem to be an expedient of doubtful propriety, unless applied in shocks of such power as to impair or destroy the irritability of the muscular fibres, and thus occasion such a relaxation as may permit a free flow of blood.

In the paralysis with relaxation, the current should be of moderate intensity, and interrupted, to secure movements, and the consequent circulation which movements induce, while in paralysis with rigidity, the current should be of overpowering intensity, and also interrupted, to paralyze the motor nerves which secure the rigidity in the voluntary muscles, and also to impair the contraction of the smooth fibres of the vessels getting their supply of nerves from the sympathetic, so that blood may flow more freely through the capillaries. By this plan of applying electricity, a relaxation of the striped fibres is secured at the same time that the capillaries are relaxed and enlarged, and, for the time being, a better circulation is secured through the rigid muscles, and an arrest or retardation of the wasting of muscular substance is realized.

In the paralysis with relaxation, without diminution of temperature, there would be theoretic reasons for employing both continued and interrupted currents of moderate intensity. The interrupted to exercise the muscles, and the continued current to give tonicity to the capillary circulation, by the tonic effect of a continuous current upon smooth muscular fibres. In all this, the chief object is to preserve the muscles from deterioration, and prevent or remove deformity, while by other means, or by the processes of Nature, the central cause of palsy may be removed, whether existing in the course of the nerves injured or diseased, or in the injured or diseased spinal cord or brain.

Brown-Sequard's theory of the action of electricity applied to muscles affected with permanent contraction is, that when applied in powerful shocks to the affected muscles, it exhausts the irritability of the muscular fibres, relieving them from the hold which the irritated nerves have upon them, and permitting them thus to relax at the same time that the smooth muscular fibres of the bloodvessels are relaxed in the same way, permitting a larger volume of blood to flow through the organs, and keeping up their nutrition until the spasmodic influences coming from the nervous centres can have time to disappear, by the restoration of the healthy functions of these centres, or by the removal of external or centripetal irritations which produce their effects by reflex action.

Brown-Sequard makes alternation of heat and cold, with the same theoretic explanation, in those conditions of parts in which sloughing occurs, especially upon the sacrum, in diseases and injuries of the spine, and in some forms of low fever. He explains that there is something more than inaction and pressure; that there is a contraction of the capillaries, which become relaxed by powerful interrupted electric currents, so that the usual amount of blood may again flow to preserve the nutrition of the parts and save them from sloughing.

Very much on the same principle, such parts are sponged with hot and cold water in rapid succession, the irritability of the muscular fibre cells of the capillaries being supposed to be exhausted by the alternation, continued for a considerable time.

3. *Inflammation*, injuring or destroying the tissues, perverting or nullifying muscular contraction, distorting and stiffening joints, and perverting, impairing, or abolishing the funtional movements of the parts affected.

Whether the inflammatory diseases which produce these results arise from wounds or injuries of the joints, or occur as the localizing of some more or less permanent constitutional condition, the indications for treatment, and the ultimate results are very similar.

The inflammations from accidents are, usually, at first synovial, attended in the beginning by excessive and thinned secretion of synovia, and afterwards by suppuration imposing upon the nervous and vascular systems violent perturbations, and involving, very soon, the surrounding tissues in inflammation, with softening and ulceration of the non-vascular articular cartilages. The processes are liable to be more rapid than is usual with spontaneous diseases of the same parts, and the cessation of diseased action, and the establishment of the permanently altered conditions earlier secured. Where, by appropriate treatment, the inflammation is aborted, before these grave results accrue, the course of the disease may be reduced to a very short period.

Spontaneous synovitis may be of two kinds—rheumatic and strumous—each more or less simple or complicated with inflammation of surrounding tissues; the former, the more painful and variable in severity, with exudations of solidifying material, which may afterwards seriously diminish the mobility of the joint affected, in consequence of roughness of surfaces, adhesions, the production of movable bodies, and the diminished flexibility of surrounding parts, the articular cartilages in some cases ulcerating, or after fatty degeneration, becoming disintegrated under the friction of the opposed cartilaginous surfaces, until the rough bony surfaces are brought into contact, to end ultimately in eburnation with surfaces polished by friction, or in anchylosis, or in such constitutional irritation as to require exsection or amputation. Where several joints are simultaneously attacked with the most acute form of this inflammation, the patient's life may be destroyed very suddenly, by the depressing influence of nervous shock.

The strumous variety of synovitis may have all grades of severity and destructiveness, and be at first, accordingly, painless or more or less painful. The easy expansibility of the synovial membrane saves it from painfulness when the changes of volume are very slow, and the involvement of the articular cartilages in destructive processes can hardly add to the pain as they are destitute of nerves, and must, therefore, be destitute of sensibility.

Whether we accept the anatomy of the synovial membrane as a tube terminating at the articular cartilage, or as closed sacks, there is no practical difference, for if the articular surface has a synovial covering, it is destitute of vessels and nerves like the cartilage beneath. This is the reason why a slow inflammation, involving destruction of parts to a frightful extent, may occasion very little pain, as long as the bony tissue escapes the inflammatory process.

Inflammation commencing in the spongy heads of the bones, must be supposed to be attended with more pain and lameness, in consequence of the free supply of nerves which are exquisitely sensitive in the inflamed state. So slow is the process in some cases, that the nerves accommodate themselves to the slowly changing volume, so as to be surprisingly free from painful sensations. From this it follows that, in a great number of cases, the diagnosis must be exceedingly obscure as to whether the soft parts or the bones have been originally attacked. It is fortunate that this distinction is of no practical importance, as the treatment of one location of disease is equally appropriate to the other. This distinction, however, may be considered clear, that when, in the course of a joint inflammation, there comes a great aggravation of painfulness, with spasmodic muscular contractions, especially aggravated by motion of the joint, or pressure of its articular surfaces against each other, the bony tissue has become involved in inflammation, and, perhaps, uncovered by the disintegration of its inverting cartilage, so as to expose its rough surface to friction.

After the subsidence of the acute symptoms in strumous synovitis, and in the earlier stages, in cases in which the initiation of the disease is by slow and doubtful steps, the pain is of the character of *aching*, increased by the dependent position. Perversions of sensation sometimes exist, as of cold or heat, when there is really no change of temperature.

In the second stage, after weeks or months, the pain is *gnawing*, with soreness referred to the bone and starting of the limb at night. In this connection, it is proper to quote the emphatic language of *Barwell on the Joints, p.* 136, where he says:—

"These sensations have been supposed to be caused by an ulceration process going on in the cartilage, and so indeed they are, but only in a secondary manner, for the pain is directly produced by the hyperæmia of the sub-articular vessels of the bone produced by the cartilaginous inflammation. Cartilage, whether healthy or diseased, possesses no nerves, the only conductors of impressions to the brain; then it is as insensible when inflamed as when not inflamed; but hyperæmia produced thereby, in such an unyielding structure as bone, sets up these painful symptoms."

"Another sensatiom, attributed with equal want of precision to ulceration of cartilages, is tenderness on pressing joint surfaces together. The origin of this symptom, although obscure, I believe myself to have detected. By questioning, for years past, every patient that came in my way; by observing the species and succession of different sensations, and examining, when possible, the joints of those whose symptoms have been thus noted, I have come to the conclusion that this tenderness indicates that the articular lamella has given way over a larger or smaller extent, and that the cancelli are laid bare to the joint."

Again, *p.* 244. "Pathology shows us that in a synovitic disease no special action is produced among the muscles, until the bone *underlying the cartilage* becomes affected. Again, we see that when *that portion* of bone is *primarily* diseased, the spasms of the muscles producing the start and shock are among the earliest symptoms. We find that a carious state of this portion of bone is extremely irritating, and sets up not merely temporary spasms, which pass like electric shocks over the limb, but that a slower and lasting contraction takes place. This phenomenon affects nearly all the muscles moving the lower bone of a diseased joint, but it predominates in the flexors, and, therefore, the lower bone becomes rigidly bent upon the upper; the muscles feeling tight and cord-like under the skin. Such contraction is produced by a morbid form of reflex action, carried from the nerves supplying the part to the muscles. * * * This contraction continues during sleep, and is of greater power and duration than any voluntary contraction. The spasms are more violent when the will is withdrawn, and they precede the paroxysms of continued pain; and the muscles affected with this peculiar contraction waste with more rapidity than in any other disease, except in certain cases of irritation of the spinal cord producing spasmodic muscular contraction. * * * Although the muscular phenomena are originally produced by the irritation of the joint disease, they eventually increase, or altogether support its morbid actions, by forcing one tender bone surface against another. * * * Sometimes, but at a later stage, when the tonic contraction of the muscles produces dislocation, the spasms and starts abate very much indeed, or disappear altogether—the displacement of one bone upon the other giving instant relief—a proof in aid of the fact (inference) that it is the mutual pressure which produces the whole train of symptoms."

Another class of joint destructions arises from immobility of the parts, with the ordinary pressure occasioned by the tonic contraction of the muscles.

Mons. BONNET, of Lyons,* quoted by Dr. H. G. DAVIS, in *Transactions of the American Medical Association*, 1863, gives the result of observations by himself and TESSIER, on the effects of prolonged immobility of joints. He says, "I am about to demonstrate, anatomically, that long continued immobility can produce severe disease in healthy joints," and then goes on to give the following results:—

1st. Effusion of blood and serum in the articular cavities.

2d. Injection of the synovial membrane, and the formation of false membranes.

3d. Alteration of the cartilages.

4th. Anchylosis.

He says, "I have not mentioned stiffness of the joints, as among the anatomical lesions which immobility produces. This stiffness is frequently observed, and ought to be particularly considered as an effect of the alterations which the autopsy reveals in the cartilages and in the synovial membranes."

Mons. TIESSIER has the credit of having first noticed the effects of immobility upon the joints. The latter says, "I have almost invariably found in all the articular cavities of the diseased limb, even in those most remote from the solution of continuity, the secretion of synovia replaced by bloody serum, and even by liquid blood almost without admixture. In one case, and one only, I have found clots of blood. This was in an old man, confined six months for fracture of the neck of the femur."

BONNET says sanguineous effusion and injection of the synovial membranes are the two first effects produced by immobility. In all the cases in which Mons. TIESSER observed them, they were already supplied with vessels, and adherent to the cartilaginous surfaces.

"Their existence appeared to demonstrate that long continued repose can produce, in the joints, lesions of an inflammatory nature."

"Continued repose can produce redness, swelling, softening, erosion, and wasting away of cartilage. The redness may be

* Traite des Maladie des Articulations, accompagne d'un Atlas, avec 16 planebes, par A. Bonnet, Prof., etc., etc. Paris et Lyons. 1845. Tome 1, f. 9, et seq.

uniform or punctate. Where the cartilage is not eroded, it presents itself under the form of ecchymosis, more or less deep. On the contrary, where the cartilage is eroded, it is unequal, dotted."

Dr. DAVIS, in the same paper, *p.* 158, quotes from Dr. WILLARD PARKER, of New York:—"I have often seen in the knee-joint, after amputation, when the joint was opened, that when the surfaces had remained long in contact, the synovial membrane and cartilage were removed by absorption, and the bone, at the same point, dead for from an eighth to half an inch in depth.

"In exsection of the knee-joint, on opening the cavity, I have found the same destruction to have occurred.

"The same pathological condition is observed in the hip-joint. Indeed, I regard it as established, that if the surfaces of joints be allowed to remain long in a fixed position, the pressure from the muscles causes destruction of the substance making the wall of the joint. We see the same condition resulting in a joint, that happens when pressure is allowed upon the heel in the management of a fracture, viz., ulceration and sloughing."

Dr. WM. H. VAN BUREN, of date 1860, in a letter to Dr. DAVIS, says, "In thinking over the many cases of diseased joints which I have examined after amputation, and otherwise, my impression is, that the greatest amount of disorganization has existed when the opposed surfaces of the joints have pressed against each other. * * * A recent case, in which I exsected the knee-joint of a young woman, for chronic strumous synovitis, afforded strong and indisputable evidence on the point in question. In the centre of each of the articular depressions of the tibia, I found a plate of necrosed bone, each about the size and thickness of a dime, lying loosely upon a bed of granulations. The articular surfaces were also profoundly altered, but except at these points not beyond the possibility of repair to the extent of anchylosis."

In this connection, it is proper to say that, through the politeness of Prof. EDMUND ANDREWS, of Chicago, I have been shown a specimen of inflammation of the knee-joint, exsected by this gentleman, in which there was an exfoliation, half an inch in diameter, upon one of the condyles of the femur, and a corresponding necrosis, preparatory to exfoliation, upon the opposed surface of the tibia. These were at points where the erect posture or the straight position would bring the greatest pressure.

It has been claimed that the occurrence of necrosis upon a joint surface, induced a similar disease in the corresponding point of the surface of the opposite bone. The light in which the subject is here viewed, renders it probable that pressure is the cause of necrosis in both bones, and that the necrosed portions are opposite each other, not because one produces the other, but because this situation is necessary to the mutual pressure of the joint surfaces, and the observed fact is that the necrosis generally takes place where the shape of the bones renders the pressure the greatest.

Dr. DAVIS quotes two cases from BONNET, of fracture of the neck of the femur within the capsule, in which (the patients dying after long confinement) all the joints below the injured joint were in a state of disorganizing inflammation, while in the joints injured, no such result was found. Dr. DAVIS explains the mystery thus:—"The head of the femur broken off and lying loose, does not feel the force of the muscular pull—the other articulating surfaces do."

A painful inflammation of joints, however excited, at length induces cramps or spasms of the muscles passing the affected joint, which may be both paroxysmal and permanent, sometimes acting with painful exaggeration, but pulling constantly, with an increase of the natural tonicity; the stronger flexors usually gaining upon the weaker extensors, until the joint becomes so flexed as to relieve the inflamed joint surfaces from the pressure at first occasioned by the muscular contraction.

The effect of this permanent spasm of the shortening muscles, is to produce a rigid state, to which BARWELL has recently given a new name, that of "contracture." This is his own definition of the condition. *On the Joints, p.* 315:—

"The muscles which are affected with contraction, gradually shorten organically and permanently; they become passively

*contractured*, that is to say, their decrease in length is not merely a passing state, which will disappear when the stimulus ceases. They become fixed in this shortened condition, either by the gluing together of their elements, or some like cause, and they cannot of themselves resume a relaxed and lengthened position. * * * It appears to me that the change is located in the sheath of the fibres, rather than in the fibres themselves. Every fibre of a muscle is composed of a sarcous and of an investing wall; the active contraction of a muscle is produced by the shortening of the flesh; passive *contracture* supervenes after the interior has been for some long time in this shortened condition, when the investing part adapts itself permanently to that shape, and each wall of every muscle cell is fixed in its abbreviated form. Moreover, each portion of areolar tissue investing the fibrous bundles assumes permanently the new form impressed upon it by the inclosed contracted sarcous. Such change does not forbid continuing active contraction, for the state, *contracture*, depends upon the change in the passive parts of the organ, to which ordinary muscular action may be added."

*Treatment.*—In the first stages of any disease of a joint involving the synovial membrane and articular cartilages, the first indication, obviously, is to give the parts the conditions of the least possible excitement or irritation. Rest, and such moderate extension as to counteract muscular contraction, measured by the feeling of comfort, meet the requirement. Warm or cold applications, according to the amount of heat to be overcome, and in violent inflammations, with rapidly accumulating pus within the synovial membrane, free, that is, long incisions into the cavity of the joint should be made, to give free outlet to the offending fluid. One of the valuable contributions to surgical science, by the late lamented Dr. E. S. COOPER, of San Francisco, was a more clear and emphatic enforcement of this expedient in acute inflammation of joints, from disease and injuries. The incisions should be free enough to discharge not only pus, coagula, concretions and sloughs whose presence, in a decomposing state, poisons the parts, aggravating the inflammation, and increasing the amount of destruction. After free incisions it is practicable to wash out the materials that do not readily flow of their own accord. The old-fashioned way of "lancing" a joint, is altogether inadequate. It is hardly necessary, however, to say that the heroic incision need not be practiced in anticipation of the active and destructive inflammation, but only after the unequivocal establishment of suppuration; for, by the proper sealing of the wound, absolute quietude, arterial sedatives, and regulation of the local temperature, with the necessary depletion by the alimentary canal, the dreaded inflammation may often be prevented. Later in the case, local stimulants, as mercurial ointment, liniments, affusion of water, and passive exercise, and then active exercise practiced during sufficiently brief periods, close the treatment.

To extend or break up adhesion, and remove distortions, apparatus must be employed, to be noticed further on.

The constitutional treatment cannot differ from that of diseases of other parts presenting the same pathology. The principles are pretty well explained by ABERNETHY, in his little work, entitled *Surgical Observations on the Constitutional Origin and Treatment of Local Diseases.* The chief advance upon ABERNETHY, is in the employment of iodine, a great amount of which, however, is thrown away, through the neglect of that eliminative treatment, without which the correcting effects of iodine and its compounds will too often fail to be realized.

BARWELL, in his work, *On the Joints*, reproduces ABERNETHY'S principles, and, in addition to them, he makes a very important distinction between the treatment appropriate to those cases of strumous diseases in which the blood fills the capillaries, producing a considerable degree of redness of the general surface, and those cases in which there is pallor and emaciation. The former cases require purgatives before iodine, iron, or other alterants and tonics will produce any abatement of the local diseased activity. It is often surprising how rapidly a local disease will ameliorate, after a thorough purgative, acting upon all parts of the intestinal tube and tributary glands, to remove secretions and excrements too long retained. The repetition once a week, for a child five or six years old, of a combination of a grain of calomel, a-twelfth of tartar emetic, and half-a-grain of compound extract of colocynth, or some similar purge, will often work wonders, after an entire failure in the employment of iodide of potassium, iodide of iron, quinia, and cod liver oil, without this purgative to precede and interlude the alterant and tonic treatment.

In inflammations of the more acute kind, much is gained in the early periods by securing, temporarily, a decided control over the circulation, and for this purpose, no agent, yet known, is equal to veratrum viride. During the depression of arterial action, the capillaries regain much of their contractility.

Blisters, setons, issues, moxas, and cauteries, actual and potential, if used at all, should be employed in the later periods of the disease. They have all fallen into great neglect of late, apparently from the present epidemic influence of sugared medicine. The pain of the application of moxas and cauteries, may be altogether neutralized by ether or chloroform, and still they are in great disfavor. It is difficult to conceive that the confidence reposed in them half a century ago, was altogether a mistake, though it was, perhaps, too great and too indiscriminate. There should not only be a freedom from activity of inflammation, but the location should be far enough from the seat of disease not to subject the near branches of the same nerve to irritation. The power of burns and wounds, and of diarrhœa from irritability of the mucous membrane of the alimentary canal, to produce those palsies, both of motion and sensation, now denominated reflex, is a presumption in favor of the power of counter-irritants to change or allay the painful excitement of diseased joints, and attendant muscular spasm. To discriminate the cases in which to employ the agency, is the point. Perhaps the most powerful and least painful of all these, is the actual cautery, applied very rapidly and very lightly, and frequently repeated. And yet the agent has gone into almost entire disuse.

BARWELL quotes a case from RUST, of Vienna, of a young gentleman suffering severely from hip disease, whose parents could only persuade him to undergo the application of the hot iron by promising to take him to the theatre that evening. The application was freely made, and the boy's pains were so much diminished thereby, and he was so cheerful, that he insisted on the fulfilment of the promise, and he greatly enjoyed the entertainment.

The influence of the counter-irritant on the disease may, perhaps, have been overestimated, by its power in paralyzing the spasmodically excited muscles, and thus diminishing the pain of their contraction.

There comes a period in the process of suppuration, when the pus has escaped from the joint, in which the propriety of opening the cavity and discharging the contents becomes a question. I incline to the advice of SOUTH, who says, in speaking of abscess of the hip-joint (*South's Chelius, vol.* i., *p.* 299), "On the whole, I think it preferable not to meddle with abscesses of the hip-joint, unless they excite much constitutional irritation, and until the skin is on the point of ulcerating; then they may be punctured, and untoward symptoms rarely follow."

It is appropriate here, to notice, more in detail, the history and philosophy of extension, as an element of treatment in inflamed joints. The earliest application of extension during the period of inflammation, which I can find, is by BRODIE in his *Pathological and Surgical Observations on Diseases of the Joints*, *p.* 145, (published about 40 years ago,) and quoted in *South's Chelius*, *American Edition*, *p.* 296, where, speaking of disease of the hip-joint, he says:—"At a later period, when, in consequence of extensive destruction of the articulation, the muscles begin to cause a shortening or retraction of the limb, I have found great advantage to arise from the constant application of an extending force, operating in such a manner as to counteract the action of the muscles. For this purpose, an upright piece of wood may be fixed to the foot of the bedstead, opposite the diseased limb, having a pulley at the upper part. A bandage may be placed around the thigh, above the condyle, with a cord attached to it, passing over the pulley and supporting a weight at its other extremity. I will not say that the effect of such a contrivance is to prevent the shortening of the limb altogether, but I am satisfied that it will, in a number of instances, render it less than it would have been otherwise, at the same time preventing, or very much diminishing, that excessive aggravation of the patient's sufferings, with which the shortening of the limb is usually accompanied."

This quotation should be regarded as remarkable, on account of the length of time the suggestion of extension seems to have lain dormant. It is remarkable that BRODIE himself, finding the expedient effective, in diminishing the painful contractions of the muscles in the later stages of the disease, did not think of using it to prevent the conditions upon which these painful contractions depend.

Dr. LIONEL BEALE, in his work *On Deformities*, published soon after *Brodie's Observations*, speaking of contractions of the knee, *p.* 99, says:—"Gradual extension, friction, the application of vapor, and mechanical aids to allow of exercise, are the means to be employed. *But, before having recourse to them, we must be certain that all active mischief has ceased, and be very cautious that we do not reproduce it.*"

It is plain that BEALE did not understand the expedient of quieting the irritation of inflamed joints by extension.

Coming down to 1860, we find BARWELL, in England, in his word, *On the Joints*, *p.* 144, (speaking of local treatment,) saying:—"The first and most important part of the local treatment, is rest. A time arrives, as we shall see, when it becomes a grave question whether entire immobility should or should not be continued, but there can be no doubt that at first the joint should be kept perfectly still by bandaging a well-padded splint upon the limb. The joint itself must be left uncovered by the bandage, for the application of any remedies that may be desirable, which in this stage of the disease belong chiefly to the class counter-irritants and derivatives."

At this date, then, BARWELL applied splints in the earlier stages of diseased joints, to secure rest, and not to produce extension and separation of adjoining surfaces, with reference to the avoidance of the irritation of the pressure of inflamed surfaces by muscular contraction.

In America, Dr. H. G. Davis, in *Transactions of the American Medical Association* for 1863, *p.* 150, says:—"I insisted from the first, on the fact (principle) that mobility is natural to, and required by, a diseased, as well as a healthy joint, and introduced that as one of the principles of my treatment.

"I insisted, also, from the first, upon the fact (principle) that pressure, mostly owing to muscular contraction, is the most active agent of destruction in the morbid process, which it is the object of my treatment to overcome, and I therefore directed my efforts to obviating, *in all the stages of the morbid process*, the pressure to which the parts diseased are exposed. To attention to this, I ascribe my main success.

"The distinctive principle of my treatment, is the procuring to the diseased structures support without pressure, and motion without friction.

"The treatment itself, concisely stated, consists in abstraction of the joint affected, by continued elastic extension."

In a paper published in the *American Medical Monthly*, 1856, Dr. Davis draws the following indications for mechanical therapeutics:—

"1. That in all diseases of joints without the capsular ligaments, extension should be applied when the joint becomes functionally immovable.

2. In diseases within the capsular ligament, the extension should be applied at once.

3. In immobility, from whatever cause, the limb must either be frequently moved, or it must be extended."

There comes very soon, in violent inflammation, a preponderance in the pull of the flexors, over that of the extensors, as if for the purpose of placing the joint surfaces in such relation to each other as to relieve them from pressure. The good result of Nature's therapy is secured by a position of the limb which renders it afterwards usless, unless a surgical process is resorted to, for the purpose of restoring the limb to the straight position. I cannot enforce this point better, than by quoting from *Tamplin on Deformities, American edition*, 1846, *p.* 168, this clear statement:—

"During inflammation (of synovial membrane) there is a constant effort to keep the joint at rest, the slightest motion occasioning the most severe pain; and the position in which there is the least amount of pressure on the articular surfaces, is undoubtedly the flexed position. Be this pressure ever so slight, it must increase the inflammation in the synovial membrane, and, consequently, the pain, which is at times most acute, requiring the most active measures for relief. The flexors, therefore, are constantly acting, and become eventually contracted, from the flexed position being maintained during the inflammatory attack."

This pathology should have suggested extension, but it did not. TAMPLIN accepts the anatomy of a synovial membrane over the articular cartilage, with sensitive nerves in it, which is a mistake. The pain from pressure at a period so early, is probably from the influence of the pressure upon the inflamed bone immediately subjacent, in which the nerves sensitive to pain have been aroused into activity.

The force to be applied, and the pain to the patient, is far less to prevent this deformity, at the same time that the surfaces are relieved from pressure upon each other, than that which is required to overcome the deformity, when once it has occurred.

Extension in inflamed joints, so far from being painful, is, on the other hand, positively grateful to the patient; so that the sensation of comfort may safely be made the test of the amount of extension required.

An instance, illustrating this, occurred to the writer, in the case of an acute traumatic inflammation of the knee-joint, with extensive suppuration and burrowing of pus, along the course of the muscles. This patient came under my care, at the end of the sixth week from the time of the accident, with a flexure of the knee to an angle of 45 degrees. Adhesive plasters were placed upon the leg, as in the treatment of oblique fracture of the femur, and extension made by a twisted rope passing from the loop of plaster under the sole of the foot, to a point eighteen inches beyond, while counter-extension was made from the chest, by the attachment of long and wide adhesive slips, before and behind the chest. Extension proved not only a comfort to the patient, but an adequate means of restoring the limb to nearly a straight position, though some degree of false anchylosis followed the extensive cicitrization of altered or destroyed parts. The turning of the cross-stick, to twist or untwist the rope, was left to the wife of the patient, who, under his direction, relaxed or tightened up the extension.

The amount of movement of one bone away from the other, is necessarily limited by the length of the articular ligaments, which, in their normal state, are adapted to hold the bones in very near proximity. If, therefore, the attempt is made to regulate the extension by any measurements, there will be a strong temptation to employ too much force, producing a painful and unnecessary tension of the ligaments holding the bones of the joints in proximity. Where the limb has already become flexed, the amount of extension which would have been comfortable during the preceding inflammatory process may be safely exceeded, but while the limb has not yet lost its straight position, the extension should not be permanently practiced to the extent of painfulness.

Barwell fully endorses this treatment, in the confirmed stage of inflammation, though he fails to advise it in the very beginning. Quoting from *p.* 328, he says:—"We have only to prevent the muscular spasm from pressing the two portions of bone together, and the disease will decrease. * * * The muscular contraction which pulls the thigh up must be met by another which pulls it down. We cannot, nor do we wish to separate the bones, but we can so arrange that the muscular force shall expend itself upon an external object, and leave between the head of the thigh and the acetabulum no more than the normal amount of pressure, perhaps less. * * * These medns will not cure the disease, but they will place it in the best possible circumstances for getting well."

*Distortions and Stiffenings after the Subsidence of Inflammation.*

The distortions and stiffenings which result from cicitrization and anchylosis, after extensive destruction of the tissues of joints, become the most intractable cases. Restoration of motion and of form, by gradual extension, is discouraging, from the slow nutritive changes of the ligaments, the cicatricial tissues, and the "contractured" muscles; and the sudden rupture, by violent force, may excite an unwelcome inflammation, or be itself altogether impracticable without tearing open the integuments, or fracturing the bones, or both, which might involve dangerous nervous shock or subsequent constitutional sympathetic irritation.

The following illustration of a tibia partially dislocated backward upon the femur, and permanently flexed, from inflammation of the joint, and the disproportionate pull of the flexors, which arises from irritation, affords a good conception of a class of irremediable deformities. Yet it would have been simple and easy to have entirely prevented this flexion and dislocation, by extension, applied before the deformity commenced. The extension would not only have prevented the deformity, but it would have diminished the inflammation and the muscular contraction, by lessening or suspending the irritation arising from the contact and frictional movements of the opposed joint surfaces.

From TAMPLIN, illustrating flexor of the tibia with displacement:—

*a* external ateral ligament.
*b* ligamentum patellæ.
*c* femur.
*d* tibia.
*e* fibula.

Fig. 2,

Sometimes, however, cases, apparently intractable, yield to mechanical force, proving abundantly successful. I quote in point, an instructive case, also from *Tamplin, p.* 169:—

"In one case," of false anchylosis, "which arose from a cart-wheel passing over the thigh, just above the knee, there was motion, but of an elastic kind, and, after the leg had been forcibly extended, it returned with an impulse to its contracted position, without the flexors acting in the least, so that it was evident there was some adhesion in the joint. The flexors I divided, however, and the extension was kept up for some weeks, with but little benefit, and, from the severity of the increasing forcible extension necessary, the patient suffered so much that he left the institution, determined to submit to no more treatment. But, after a few weeks, he returned penitent, when I again operated, and resumed extension. We progressed, as heretofore, very slowly. From the constant pressure kept up on the anterior part of the thigh, a slough made its appearance, upon the healing of which I resolved to make no more attempts, and informed the patient that I feared nothing further could be done for him. This frightened him, and he set to work to screw his leg straight, at all hazards, upon doing which, a sudden and loud snap was heard, compared by the other patients to the report of a pistol, and immediately the leg could be placed in a straight position. * * * He has left with a comparatively useful limb."

The division of tendons, and such ligaments as can be reached, may greatly aid the effort at restoration, and, perhaps, if there is any degree of motion, the attempt should be made to convert useless limbs into useful ones, for, after the failure of strenuous efforts, the patient is apt to be more reconciled than before.

The division of the muscles themselves, may sometimes greatly aid in the restoration of the proper position. In the vicinity of large arteries, however, the subcutaneous division is not free from danger, however skilled the operator may be in anatomy, for there may be deviations from the usual relations.

That so accomplished an operator as Dr. SAYRE, of New York, should have lost a patient from wound of the femoral artery, under these circustances, is justification enough for this caution. See *Transactions American Medical Association*, 1863, *p.* 168.

In bony anchylosis, or in false anchylosis, in which the ligamentous joining resembles that of an ununited fracture, the only remaining resource is Barton's operation, which consists in sawing the bone and removing a piece of such a shape as to permit the restoration of the limb nearly to its original general direction. This operation was originally practiced by Dr. BARTON, of Philadelphia, upon the lower portion of the femur, and has since been repeatedly practiced.

This operation was performed upon the femur, above the trochanter minor, by Dr. L. A. SAYRE, of New York, in 1862, making a movable and useful joint; a tolerable substitute for the hip-joint, which had become motionless from anchylosis. See *Transactions N.Y. State Medical Society*, 1863, *p.* 103.

4. *Accidents*, breaking, tearing, bruising, burning, or freezing the tissues, followed by the loss of the parts, or the failure, imperfection, or perversion of their union or restoration.

The deformities resulting from breaking the bones and tearing the ligaments, are more easily prevented than removed. The accidents are sometimes of such a nature as to render some degree of deformity unavoidable, but in those cases, much may be done, by skilful management, to obviate permanent malpositions and ill-shapes.

The discussion of the treatment of fractures and dislocations, is outside of the scope of this paper. Some of the deformities after fractures admit of great amendment, by bending the new formed bone when the deviation of the long axis has occurred, from neglect of extension or lateral support, or from premature use of the limb, and the remedy is not delayed too long; and at a later period, by refracturing the bone and keeping the fragments in proper relation to each other, during the period of repair; or by drilling the bone to secure inflammation and softening, as suggested by Dr. BRAINARD, in *Transactions American Medical Association*, 1854, and, after about ten days, employing force, applied gradually, to secure a change of nutrition with the changing shape, or applied suddenly, to overcome the deviation by interstitial fractures.

After severe bruises, with or without laceration of parts, the destructive inflammation and gangrene, with extensive sloughing of intermuscular tissue, in the progress of areolar erysipelas, the greatest attention to passive movements may be necessary, during the period of repair, to prevent the formation of permanent adhesions and malpositions, and to stimulate the absorption of the organized exudations, before they have assumed such density of structure as to render them extremely difficult of elongation. In consequence of this, it may be necessary to divide them, in order to secure the proper restoration of position; and, where the integuments are involved, to perform plastic operations, to cover the affected parts with healthy integument.

After freezing, the indication is to recure sufficient slowness in the thawing of the parts, by the employment of snow or cold water. The sloughing of integument may, in some cases, result in cicatrices, resembling those from burns, and requiring similar treatment, both for prevention of deformity and its correction.

Burns result in some of the most frightful distortions, exceedingly difficult to prevent by extending appliances, and more difficult to remove by any means except the excision of the cicatrix. It is surprising to see into what a minute space a cicatrix will contract, when resistance to its contraction is removed.

The true indications in the treatment of burns, with reference to the prevention of deformities, are to moderate the intensity of the inflammation, first, by the local employment of turpentine or other capillary stimulant, and the internal administration of opiate and alcoholic remedies, to counteract shock and to annul or diminish reflex action; then, to exclude air and other local irritants, by the application of flour, eggs, lead-paint, poultices, &c., and to control the general circulation by alvine evacuants and veratrum viridi, or other arterial sedative; and, finally, with the commencement of granulation and cicatrization to support the general powers by nourishing food, and tonics if need be; and to counteract the drawing of the cicatrix by mechanical appliances. By this means, the shortening of cicatrices on the limbs, will stretch the sound skin chiefly in the direction of the circumference; and when the contraction of the cicatrix has become complete by the yielding of the skin in this direction, the danger of distortion from the longitudinal pull is chiefly past.

To elongate a contracted cicatrix, in order to remove distortions already produced by its contraction, is altogether impossible. The correct treatment is, to remove the cicatrix, and supply its place by sound skin, taken from neighboring parts, by a plastic operation; and, where this is impracticable, to make a free incision through the narrowest part of the contracted bridle, to straigthen the limb and hold it in its restored position, until, by the contraction of the new cicatrix, the sound skin has been drawn in from other directions. In some cases, an elastic retention will resist the pull of the cicatrix, at the ame time that the functions of the muscles and joints are preserved, and the condensed sub-cicitricial tissue is more readily loosened up by the to and fro movement, and the surrounding integument is more speedily and more perfectly enlarged, to compensate for the contracting cicatrix. A rubber spring is most convenient for this purpose. Where the situation is not upon the limbs, but upon the neck, chest, or abdomen, the resistance to the contraction of the cicatrix is more difficult, and is sometimes impossible. Adhesive plasters, into the middle portions of each strip of which a portion of a rubber ribbon is inserted, promise more than any other expedients. The plaster must inevitably slide somewhat, and the rubber will secure an efficient pull, notwithstanding the sliding, and that, too, without so much interference with the ordinary movements as plaster alone imposes.

5. Mutilation of parts, designedly done, or force or restraint, artificially applied, like the compression of the feet of the female children of Chinese grandees, the heads of some American Indian tribes, and to a less degree, the feet and waists of genteelly educated children in modern American and European society.

We laugh at the tastes and usages of people who attempt to secure distinction for themselves and their children, by compression and mutilation of various portions of the body, so that a lady has the inimitable distinction of requiring to be carried everywhere she goes, because her feet have been purposely made useless to her; a chief, the high honor of having a head shaped like an idiot's, or the face slit in various parts for the insertion of trinkets. But it may justly be claimed, that these exhibitions of taste are reasonable, compared with the practice among us of compressing and staying up the chests of growing girls, so as to diminish the capacity of the lungs, and so weaken the spinal muscles as greatly to favor lateral curvature.

A strange sentiment prevails among us, that narrow feet indicate refinement, and broad feet, vulgarity; and so, forsooth, every mother, for her children, and all vain youth of both sexes for themselves, must compress the feet with shoes too narrow for their size, until a portion of the toes ride upon the others; and the whole foot is so compressed as to fail of the graceful elasticity natural to it. Not the least deplorable condition of the deformities resulting from these usages, is the impossibility of removing in the adult the misshapes acquired in childhood and youth. A deformity, admitted to be such, may be subjected to treatment early; but a deformity, deemed fashionable, will receive no attempt at remedy, until suffering prompts the victim, too late, to seek relief.

There is one possible good of these deformities; that is, to convince ignorant people that, as the body is capable, in the growing period, of having these abnormal shapes purposely produced; so, by appliances purposely made, deformities may be gradually corrected.

## Figures and Tables

**Fig. 1. f1:**
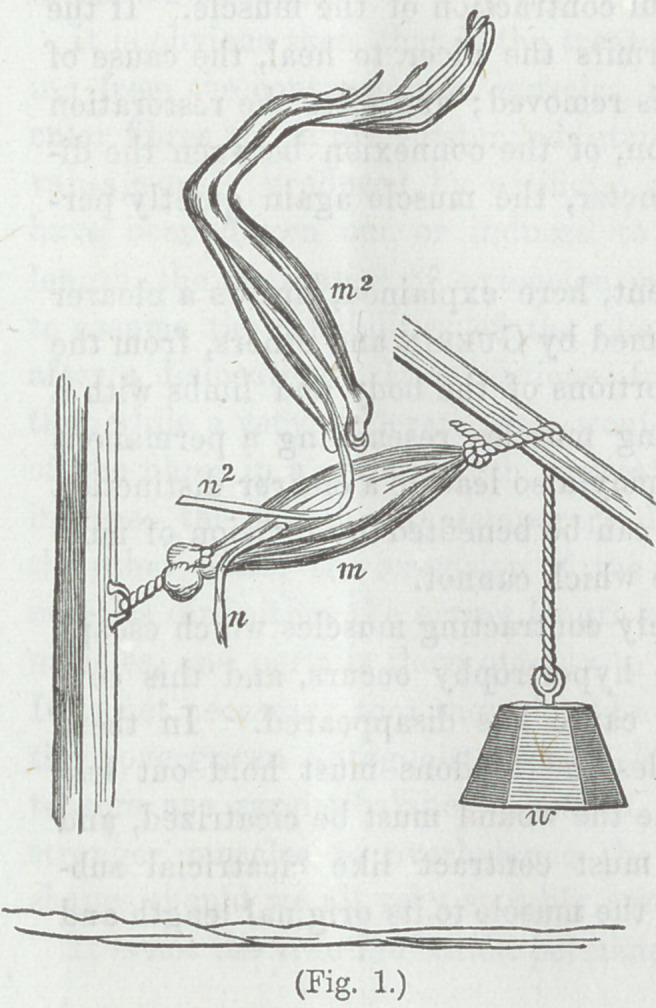


**Fig. 2, f2:**